# Supplementary material for: A Probiotic Formula for Modulation of Colorectal Cancer Risk via Reducing CRC-Associated Bacteria
Source: Cells. 2023 Apr 25;12(9):1244. doi: 10.3390/cells12091244 (PMC10177585; doi:10.3390/cells12091244)
Supplement: Supplementary file 1 [file cells-12-01244-s001.zip › cells-2211745-supplementary.pdf]

Table S1. Nucleotide sequences of primers and probes used in this study

**Primers**	**sequence (5'-->3')**
B. bifidum-F	CCTCGATGCCGTGACTGACT
B. bifidum-R	GTGAGCCAACTCCGGAATGA
B. adolescentis-F	AAGTTCGACGCCAGCAGAAA
B. adolescentis-R	TTTGCGACACAGCAGAATCG
B. longum-F	CCGTGGTGTGGGTGGTATTC
B. longum-R	TCCAACAAGTCGCCCTTGAT
Bc-F	TCCATCCGCAAGCCTTTACT
Bc-R	GCTTCCGGTGCCATTGACTA
m3-F	AATGGGAATGGAGCGGATTC
m3-R	CCTGCACCAGCTTATCGTCAA
Ch-F	GGGCTGCGGAAGCAACTTA
Ch-R	GATGACCTCGCCCTGATCAT
Fn-F	TTCAATAAAAGTGGCAGGTCAAG
Fn-R	TAACAACACATGCAGGTCAATGG
C-F	CGTCAGCTCGTGYCGTGAG
C-R	CGTCRTCCCCRCCTTCC
**Probes**	**sequence (5'-->3')**
B. bifidum	TGCCGCTGCACTCTCGTGATGC
B. adolescentis	ACTGCACGATTCCATCGCAGGAGCT
B. longum	ATTTCCCCGCGCTCGTTCGC
Bc	TTCATCATCACAGCCGACAACGCA
m3	AAGCCTGCGGAACCACAGTTACCAGC
Ch	ACCACCACACAGGACGGAAAGATTCTCC
Fn	ACTCGAACCCCCAACCCTCGGTTT
C	TTAAGTCCCRYAACGAGCGCAACCC
